# Structure of higher-order interactions in social-ecological networks through Q-analysis of their neighbourhood and clique complex

**DOI:** 10.1371/journal.pone.0306409

**Published:** 2024-08-26

**Authors:** Udit Raj, Arnab Banerjee, Santanu Ray, Sudeepto Bhattacharya

**Affiliations:** 1 Department of Mathematics, School of Natural Sciences, Shiv Nadar Institution of Eminence (Deemed to be University), Greater Noida, Uttar Pradesh, India; 2 Department of Zoology, School of Life Sciences, Sikkim University (A Central University), Gangtok, Sikkim, India; 3 School of Informatics, Kerala University of Digital Sciences, Innovation and Technology, Veiloor, Kerala, India; Scuola IMT Alti Studi Lucca, ITALY

## Abstract

This paper studies higher-order interactions in social-ecological networks, which formally represent interactions within the social and ecological units of an ecosystem. Many real-world social ecosystems exhibit not only pairwise interactions but also higher-order interactions among their units. Therefore, the conventional graph-theoretic description of networks falls short of capturing these higher-order interactions due to the inherent limitations of the graph definition. In this work, a mathematical framework for capturing the higher-order interactions of a social-ecological system has been given by incorporating notions from combinatorial algebraic topology. In order to achieve this, two different simplicial complexes, the clique and the neighbourhood complex, have been constructed from a pairwise social-ecological network. As a case study, the Q-analysis and a structural study of the interactions in the rural agricultural system of southern Madagascar have been done at various structural levels denoted by *q*. The results obtained by calculating all the structural vectors for both simplicial complexes, along with exciting results about the participation of facets of the clique complex at different *q*-levels, have been discussed. This work also establishes significant theorems concerning the dimension of the neighbourhood complex and clique complex obtained from the parent pairwise network.

## 1 Introduction

A social-ecological system (SES) is a complex adaptive system [1–3]. Understanding the intricate interplay between social systems and ecological units lies at the core of comprehending the evolution of social ecological systems. The nexus between these two domains hinges on the quantification of information exchange, a pivotal step towards unraveling their interdependency. In every such system, there are corresponding social-ecological networks (SEN) that transform interactions within the system into a mathematical format, thereby creating a formal representation of the intricate social-ecological structure [4–8].

SES contains interactions between social units (social actors) and ecological units. Most of the SENs proposed in the scholarship are based on the graph theoretic framework, which has provided very important foundational results in the study of SEN when pairwise interactions are considered. It has deep implications for the structural description of the corresponding SES, but exhibits limitations in capturing the complexity of these systems [9–14]. In the present paper, we build on this framework to include non-pairwise higher-order interactions (HoIs) of the SES using concepts from combinatorial algebraic topology to encode higher-order interactions present in the SES as simplices in the corresponding higher-order SEN. It may be noted that it includes the graph theoretic framework and goes beyond it to describe the non-pairwise HoIs in the SES when the group size of the interaction is more than two [15, 16]. We hope that our studies complement the existing studies of the structure of SEN.

As many real-life social-ecological networks exhibit higher-order interactions (HoIs), for example, the dependency of humans on forest lands leads to many complex relations between social and ecological units that are not limited to pairwise. In the context of the interaction between two social units, denoted as *S*1 and *S*2, and an ecological unit, *E*1, when both *S*1 and *S*2 utilise the same ecological patch, it establishes a social connection between them. Consequently, the entities *S*1, *S*2, and *E*1 engage in a higher-order interaction, reflecting the interplay of social and ecological elements. However, when this interaction is represented using a graph-theoretic model, certain information may be lost. The graph-theoretic model interprets the interaction as separate edges, namely {*S*1, *S*2}, {*E*1, *S*1}, and {*E*1, *S*2}. This representation fails to capture the higher-order nature of the interaction, resulting in information loss. These HoIs cannot be captured by the graph-theoretic based framework due to the definition of graphs. For a given graph-theoretic network *G*, defined on the vertices set *V*, *E* is the set of edges such that *E* ⊆ [*V*]^2^, where [*V*]^2^ is the set containing those elements of P(V), whose cardinality is 2 [17]. Hence, any interaction which involves more than two units cannot be captured by *G*. Moreover, recognising and appreciating higher-order interactions is critical for knowing the resilience, stability, and functioning of social ecological systems, as well as for designing effective management and conservation methods. In this work, to capture these interactions along with the pairwise ones for understanding the structural details, a mathematical tool from algebraic topology called simplicial complex has been used for the modeling of SES. A simplicial complex *K* is a collection of simplices (which represent the interactions of the given system) along with the downward closure property [18, 19]. If the simplicial complex *K* is defined on a vertex set *V*, then the subsets of *V* are the simplices of *K*. These simplices represents the interaction present in the system, such as 1-simplices represent the edges or the interactions between two units, 2-simplices represents the triangles or the interactions between three units of the system etc. Hence, we can conclude that graph-theoretic networks has the 1-simplices only which are edges.

**Fig 1:**
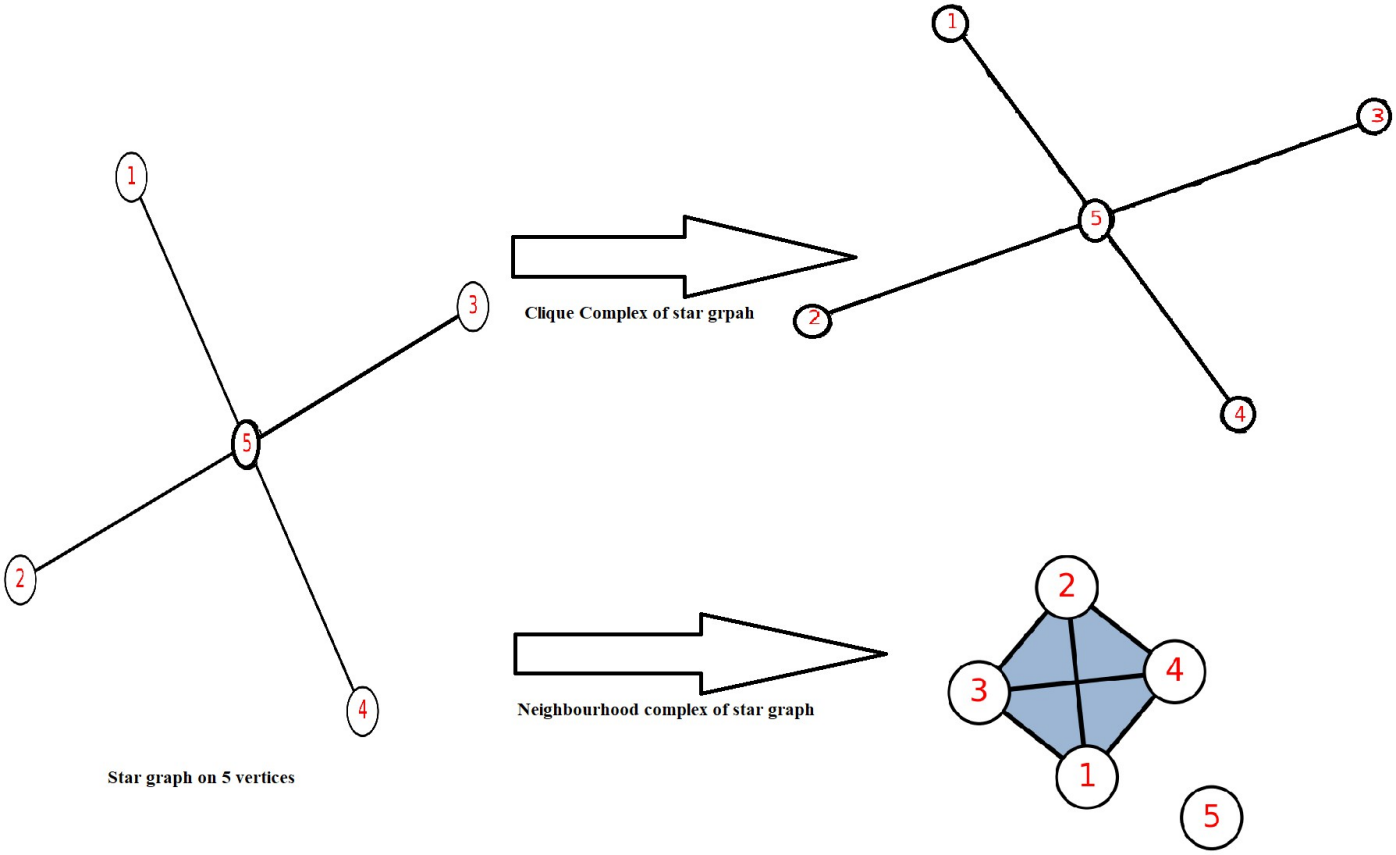
Clique and neighbourhood complex of star graph on 5 vertices *S*_5_.

A given pairwise network can be reconstructed into the simplicial complex in order to capture both lower-order and higher-order interactions. Simplicial complexes which can capture the non-pairwise interactions, can also be built directly from available data, or they can be built in a variety of ways from given undirected or directed networks (digraphs). In this work, we have constructed two abstract simplicial complexes from the available pairwise network: the neighbourhood complex and the clique complex. In the clique complex of any given graph *G*, the vertices of the complex are the same as the vertices of *G*, with the complete subgraphs (cliques) as simplices. Whereas in a neighbourhood complex, corresponding to every vertex *v* of *G*, there exists a simplex *σ*(*v*) in *N*(*G*), which consists of the adjacent vertices of *v*. An example of both the simplicial complexes from the given graph *S*_5_ is presented in Fig 1. The star graph on 5 vertices, contains 4 vertices with degree 1 and 1 vertex with degree 4. The corresponding neighbourhood complex contains one simplex of dimension 3 and four simplices of dimension 0, whereas clique complex is same as *S*_5_.

The real-life social-ecological system, which is a rural-agricultural system from southern Madagascar *N*_*M*_ has been studied by constructing its clique and neighbourhood complex from the available pairwise network. It has been adopted from the article [4], which contains 14 ecological units that are the forest patches and 8 social units that are the clans that utilise and maintain the ecological patches. The pairwise network contains the interactions between social-social units, ecological-ecological units, and social-ecological units. If two social units utilizing same forest patch then they are said to be socially connected, whereas two ecological units are related by the relation of seed-dispersal. If a clan (social unit) utilise the forest patch (ecological unit) by any means, then they are called related to each other. In order to construct a clique complex *C*(*N*_*M*_) from pairwise network *N*_*M*_, an overarching relation *R* is required by which a group interaction between the units of SES can be defined. If the size of these group interactions are more than 2, we will consider them as HoIs otherwise these interactions will be considered as lower-order interactions. The overarching relation which form the simplices is: generating and managing ecological resources in the same forest patch. The proposed relation *R* involves relationships that encompass social-social, social-ecological, and ecological-ecological interactions. Consequently, an assemblage of social and ecological entities can collectively embody a Higher-order Interaction (HoI) through the utilisation of the overarching relation *R*.

The neighbourhood complex *N*(*N*_*M*_) consist simplices *σ*_*i*_ corresponding to each vertex *i* of *N*_*M*_. Each simplex can be seen as *σ*_*i*_ = {*V*_1_, …, *V*_*k*_}, where *k* is the degree of vertex *i* in *N*_*M*_ and *V*_*i*_’s are the ecological or social units. Moreover, to better understand the structural details and topological features of the given system, Q-analysis of both the constructed simplicial complexes has also been done. Q-analysis was first used by Atkin for the study of social systems, Q-analysis of any simplicial complex allows us to study at the local, meso-scopic, as well as global levels by calculating all three structural vectors [20–23]. It is a hierarchical study in which a complex *K* can be studied at different connectivity levels denoted by *q*, where 0 ≤ *q* ≤ *dim*(*K*). At each connectivity level, simplices of dimension greater than or equal to *q* of complex *K* have been checked for *q*-nearness and *q*-connected components. As these simplices represent the interactions in the Madagascar system, they reveal interesting structural patterns of system. Q-analysis of the studied network reveals structural details about the higher-order interactions between the social actors and forest patches, which are interdependent on each other.

All three structural vectors of the constructed simplicial complexes have been calculated and discussed, which allows us to understand the structural differences in both complexes. For both the complexes, facets and their corresponding *q*-connected components at each *q*-level are given by an important result that is established in theorem 3.5. Along with this Simmelian brokerage and the topological dimension of every 0-simplex (vertices) has also been calculated. Simmelian brokerage allows us to estimate the social-ecological capital of each vertex in *N*_*M*_. Whereas the topological dimension of each 0-simplex *v*_*i*_ gives us the number of simplices in a simplicial complex that have the 0-simplex *v*_*i*_ as a face. Hence, both simmelian brokerage and topological dimension gave us the influential 0-simplices of a simplicial complex, and the important social and ecological units can be identified.

The contribution of this article is as follows: (1) Study of higher-order interactions in a social-ecological network by constructing its clique and neighbourhood complex; (2) Q-analysis of the two constructed simplicial complexes reveals deeper structural patterns of HoIs in the Madagascar social-ecological network. Moreover, using the structural vectors, the formation of HoIs and their connectivity at different *q*-levels were also analysed; and (3) Developed theorems in section 4.1, which establish a relationship between the dimension of the neighbourhood complex and the clique complex constructed from a pairwise network. Moreover, the objective of this work is to study the structural participation of simplices in any given specific social-ecological network, while the background of the work shall be entirely combinatorial.

The paper is organised as follows: Section 2 provides some standard definitions in combinatorial algebraic topology to make the article self-contained. Section 3 illustrates the construction of clique and neighbourhood complexes along with important theorems related to the dimension of complexes that have been developed in this work. In Section 4, we have discussed the Q-analysis of the neighbourhood complex of the Madagascar network. Important results and calculations have been discussed in Section 5, and Section 6 briefly concludes the work.

## 2 Preliminaries

This section provides some standard definitions in combinatorial algebraic topology, to make the article self-contained. Most of the definitions presented in this section can be found in the literature [24–29].

### 2.1 Notations

Throughout this work *N*_*M*_ represents the social-ecological network of southern Madagascar, *C*(*G*) is the clique complex of the graph *G* and *N*(*G*) is the neighbourhood complex of graph *G*, otherwise stated.

**Definition 1** *Let V be a non-empty, finite set with* |V|=n+1,n∈N *whose elements are the vertices, and are denoted by v*_*i*_, *i* = 0, 1, …, *n*. *Any member of the power set of V*, P(V), *is an abstract combinatorial object called a simplex over V*.

**Definition 2** *Given a set V as in the previous definition, a family K of simplices is called a simplicial complex if it is closed under subset inclusion, that is*:

$$\forall \sigma_{i}\in K\;\;and\;\;\left(\sigma_{j}\in P\left(V\right),\;\;if\;\;\sigma_{j}\subset \sigma_{i}\Rightarrow \sigma_{j}\in K\right).$$

By the above condition vertex set of simplicial complex *K* can be written as *V*(*K*) = {*v* ∈ *V*|{*v*} ∈ *K*}. Moreover, taking a subset of simplices of *K*, with the subset inclusion property is called subcomplex of the simplicial complex *K*.

**Definition 3** *Given a simplicial complex K, the elements of K are called simplices and the dimension of any simplex σ* ∈ *K*, *dim*(*σ*) *is defined as dim*(*σ*) = |*σ*| − 1.

**Definition 4** *The dimension of a simplicial complex K, denoted by dim*(*K*), *is defined to be r* ≥ 0 *where r is the largest natural number such that K contains an r-simplex (that is simplex of dimension r*).

A simplicial complex of dimension *L* contains simplices of every dimension starting with 0 to *L*. Each simplex *σ* ∈ *K* represents the interaction of order |*σ*| − 1, interactions are classified as higher-order if |*σ*| ≥ 3 and as lower-order if |*σ*| ≤ 2. This makes the simplicial complex a suitable mathematical object for capturing the interactions of a given complex system. Among the simplices of a simplicial complex, the maximal simplices play a very relevant role, and the sequence of maximal simplices can fully determine the simplicial complex [30].

**Definition 5** *In a simplicial complex K, for a given* p∈N , *a collection of simplices of dimension atmost p is called p-skeleton of K*.

In a simplicial complex *K*, we can always construct a subset of simplices *K*^*p*^ = {*σ* ∈ *K*|*dim*(*σ*) ≤ *p*} of dimension up to *p*, where *p* ≤ *dim*(*K*). This allows us to see a graph-theoretic network as 1-skeleton of any simplicial complex. From the given simplicial complex, one can construct a collection of all simplices of dimension atmost 1, which is the collection of all vertices and edges only. This collection of simplices will be a sub complex of dimension 1 and contain only the interactions of order 1. Hence, for a given SES, a definition using the simplicial complex that can capture the HoIs along with the pairwise interactions is given as:

**Definition 6** *A social-ecological network (SEN) is a combinatorial object and its structure is given by M*_*SES*_ = (*K*, ∧) *along with an algorithm A such that for* ∧ ≠ *ϕ*, *α* ∈ ∧ $\subset N^{+}$, *K is a simplicial complex over V, the universe of an SES, with dim*(*K*) ≥ 1 *given by the algorithm A*(*α*).

On putting *α* = 1, the algorithm *A* will capture only interactions of order 1, which are the pairwise interactions. Hence, by varying value of *α* one can capture the interactions of various orders present in the SES. Simplices of dimension two or more represent the higher-order interactions of the system, whereas simplices of dimension less than two represent the lower-order interactions. Moreover, simplices of every dimension share faces with each other, and the dimension of shared faces among the simplices is called the strength of connectivity. If two simplices share a face of dimension *m*, then their connectivity strength is *m*. More the dimension of shared faces, more the strength of connectivity.

**Definition 7** *In a simplicial complex K of dimension k, connectivity level q is defined as the strength of connectivity among the simplices of dimension q or more*.

Due to the subset closure property, any subsimplex of a simplex is also a simplex induces various levels of adjacency as well as various levels of connectivity between the simplices. These connectivity levels are denoted by *q* and 0 ≤ *q* ≤ *dim*(*K*). At every *q*-level, simplices of dimension *q* or more are checked for their *q*-nearness and *q*-connectedness. Hence, *q*-level refers to the strength of connectivity among the simplices of dimension *q* or more. At each *q*-level, strength of connectivity of two connected simplices is *q*, as they share a *q*-dimensional face with each other. In the next definition, we will see when the two simplices are *q*-near to each other.

**Definition 8** *Two simplices σ*_*i*_ *and σ*_*j*_ *of a simplicial complex K are q-near if they share a q-dimensional face and hence, they are also* (*q* − 1), (*q* − 2), …, 1 *and 0-near*.

The *q*-nearness of two simplices can be used to construct a sequence of simplices in which consecutive simplices are *q*-near to each other. Moreover, two simplices *σ*_*i*_ and *σ*_*j*_ (*σ*_*i*_ ≠ *σ*_*j*_) are said to be *q*-connected if there exists a sequence of simplices starting with *σ*_*i*_ and ending at *σ*_*j*_, such that any two consecutive simplices are *q*-near. If, two simplices are *q*-connected then they will also (*q* − 1)−, (*q* − 2)−, …, (0)− connected. A collection of such simplices of any simplicial complex *K* that are *q*-connected to each other is called a *q*-connected component of *K*. Hence, a simplicial complex can be divided into *q*-connected components.

From the combinatorial aspect three structure vectors of the simplicial complex *K* of dimension *k* are defined:

- The first structure vector *Q*_*q*_: The *q*^*th*^ entry of the *Q*-vector of dimension *k* + 1 denoted by *Q*_*q*_ is equal to the number of *q*-connected components. This vector provides information on the number of connected components at each level of connectivity with initial level being equal to the dimension of the complex: *Q*_*q*_ = [*Q*_*q*=*k*_*Q*_*q*=*k*−1_ … *Q*_*q*=1_*Q*_*q*=0_] [31].
- The second structure vector *n*_*q*_ is an integer vector with *dim*(*K*)+ 1, is given as *n*_*q*_ = {*n*_*q*=*k*_*n*_*q*=*k*−1_ … *n*_*q*=1_*n*_*q*=0_}; where the *q*^*th*^ entry, *n*_*q*_, is equal to the number of simplices with dimension larger or equal to *q*, that is, it is equal to the number of simplices at the *q*-level.
- The third structure vector $\bar{Q_{q}}$ is the simplicial complex’s global characteristic that assesses the degree of connectedness on a *q*-level. In other words, it counts the number of *q*-connected components per the number of simplices, with $\bar{Q_{q}}$ components defined as: $\bar{Q_{q}}$ = $1-\frac{Q_{q}}{n_{q}}$.

## 3 Materials and methods

**Fig 2:**
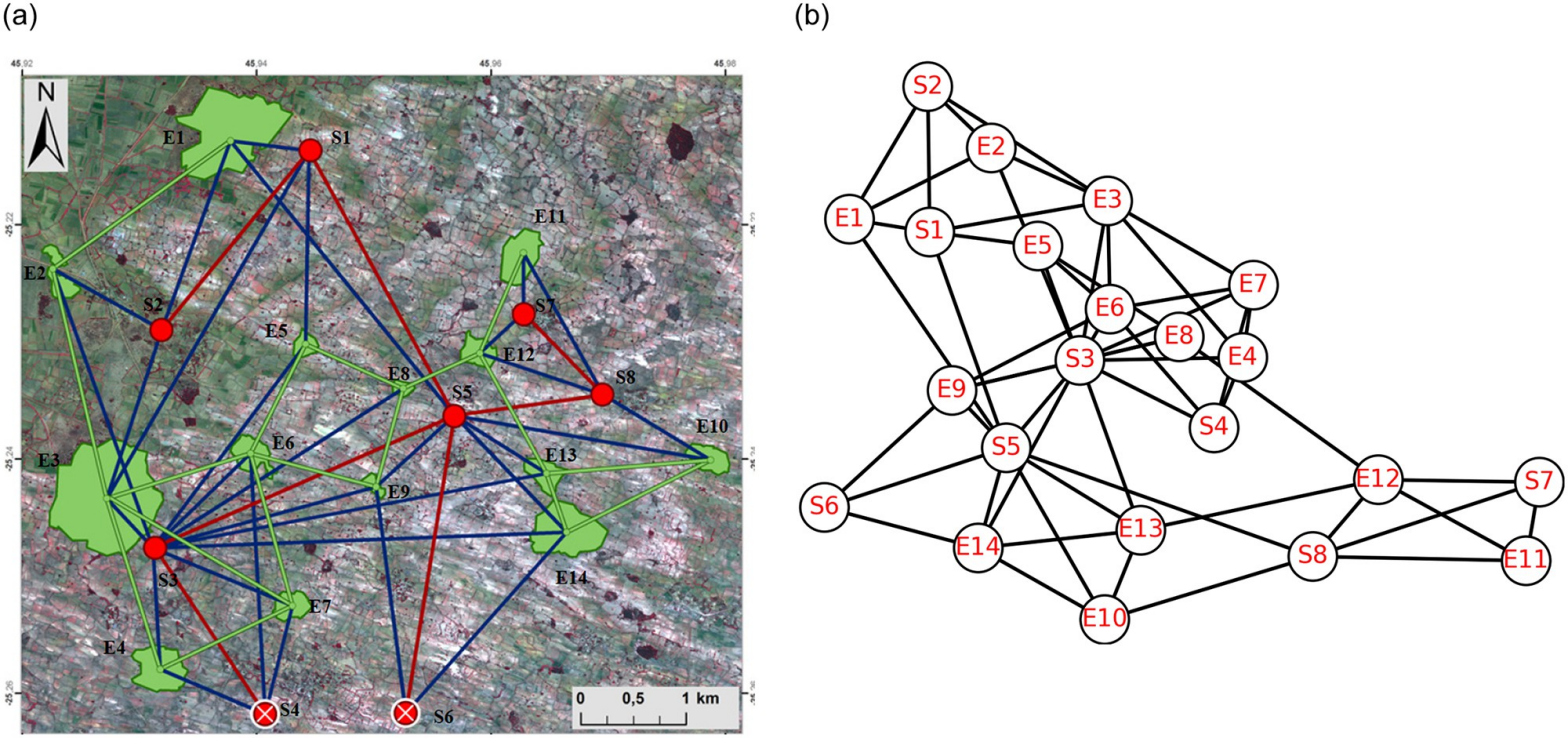
(a)* Rural agricultural system in Southern-Madagascar described as a social–ecological network [4], (b) Pairwise network representation of the social-ecological system, Ei’s and Si’s represent ecological and social units respectively.

In this work, a rural agricultural system in southern Madagascar is described as a social-ecological network represented by *N*_*M*_. It consists of 14 ecological units, which are forest patches, and 8 clans, which maintain the forest patches and constitute the social units [32–34]. The relationship that defined the edges along with the simplices in this network is “generating and managing ecological resources in the same forest patch”, with the help of this relation higher-order interactions have been defined in *N*_*M*_. For the structural analysis of this social-ecological network, the clique complex and neighbourhood complex have been constructed. Rural agricultural system and its pairwise network representation has been given in Fig 2 (*This figure has been taken from the article “Bodin Ö, Tengö M. Disentangling intangible social–ecological systems. Global Environmental Change. 22(2):430–9; 2012).

On the one hand, the neighbourhood complex of the network *N*_*M*_ is denoted by *N*(*N*_*M*_) consists of 22 simplices corresponding to every vertex *v* of *N*_*M*_, which are defined by the adjacent vertices of *v*. Each simplex in *N*(*N*_*M*_) is denoted by *σ*(*v*_*i*_), corresponding to the vertex *v*_*i*_ for *i* = {1, …, 22} (initial 14 vertices are ecological units, which are represented by *Ei* and the remaining are social units, represented by *Si*). Hence, the structure of neighbourhood complex is given as, *N*(*N*_*M*_) = {*σ*(*v*_1_), *σ*(*v*_2_), …, *σ*(*v*_22_)}, along with their subsets. For example, *σ*(*v*_1_) = {*E*2, *S*1, *S*2, *S*5} is a simplex corresponding to vertex *v*_1_ which is an ecological unit *E*1, adjacent with four vertices from the vertex set of *N*_*M*_. Similarly, all the 22 simplices have been defined. On the other hand, the clique complex of the network *N*_*M*_ is denoted by *C*(*N*_*M*_) and consist of all the complete subgraphs of *N*_*M*_. It further implies that all the complete subgraphs of size *n* of *N*_*M*_ will become simplex of dimension *n* − 1 in *C*(*N*_*M*_).

### 3.1 Clique complex and neighbourhood complex of a graph

For capturing the non-pairwise interactions of a given network *N*, using a simplicial complex, mainly two combinatorial approaches are defined in the literature [35–38]. One is a clique complex, and the second is a neighbourhood complex. Both of these simplicial complexes can be constructed by a given pairwise network *N*. The clique complex contains the complete subgraphs of *N* as simplices, while the neighbourhood complex contains the simplices as a subset of vertices of *N* that have a common neighbour. In other words, the clique complex captures the global connectivity of the network, while the neighbourhood complex captures the local connectivity of the vertices of *N*. Both of these simplicial complexes contain simplices, which represent the interactions of both types (higher-order and lower-order) in the original network *N* that cannot be captured by the graph-theoretic network *N*. So, we can get two different simplicial complexes from the given network *N*, which capture the different structural properties of this network. Additionally, they both provide a way to encode the connectivity structure of a given graph into a combinatorial object that can be analysed using topological methods.

**Definition 9** *Clique complex C*(*G*) *of a graph G defined on a vertex set V is the simplicial complex whose simplices are all subsets of the vertices that form a clique in the graph*.

**Definition 10** *Neighbourhood complex N*(*G*) *of a graph G defined on a vertex set V is the simplicial complex whose simplices are subsets of the vertex set which have the common neighbour in the original graph G*.

A *k*-dimensional simplex in *N*(*G*) is a set of *k* + 1 vertices that have a common neighbour in *G*, which can reveal information about the clustering of vertices in *G*. Additionally, the neighbourhood complex contains simplices of dimension *deg*(*v*_*i*_) − 1 for each *v*_*i*_ ∈ *V* where *i* ∈ [1, …, *n*]. It encodes the pairwise relationships among vertices as well as the higher-order relationships among subsets of vertices of *G*.

Table 1 signifies that depending upon the parent graph, the dimension of clique and neighbourhood complex can vary, which further defines the dimension of structural vectors. So a classification based on the graph type (complete or not complete) is given in the next two theorems, which helps us understand the dimension of structural vectors better. We have classified the parent graph as complete or non-complete, and based on this, the *q*-connectivity of corresponding simplicial complexes can be studied.

**Table 1:** Dimension of different simplicial complexes corresponding to complete graphs.

Complete graph *K*_*n*_	Dimension of clique complex	Dimension of neighbourhood complex
*K*_1_	0	Empty
*K*_2_	1	0
*K*_3_	2	1
*K*_4_	3	2
*K*_5_	4	3

**Theorem 2.1** *Dimension of neighbourhood complex N*(*K*_*n*_) *corresponding to complete graphs K_n_ is always less than the dimension of clique complex C*(*K*_*n*_).

*Proof*. Let *K*_*n*_ be a complete graph on *n* vertices. Then dimension of *C*(*K*_*n*_) = *n* − 1, as every clique of size of *n* is a (*n* − 1)-simplex in its corresponding clique complex. Now, in *K*_*n*_ degree of each vertex is *n* − 1, which further implies that set of these *n* − 1 vertices will form a (*n* − 2)-simplex in the neighbourhood complex *N*(*K*_*n*_).

Let assume that the dimension of neighbourhood complex *N*(*K*_*n*_) is *n* − 1, implies that there exists a (*n* − 1)-simplex *σ* = {*v*_0_, *v*_1_, …, *v*_(*n* − 1)_}.

⇒ All the vertices of *σ* have a common neighbour in the original graph *K*_*n*_, let say *v*. These *n* vertices together with *v* belong to *K*_*n*_ which is a contradiction. Hence, dimension of *N*(*K*_*n*_) is always less than dimension of *N*(*K*_*n*_).

**Theorem 2.2** *For a given non-empty connected graph G defined on vertex set V of cardinality n, if G* ≠ *K*_*n*_ *then dimension of clique complex cannot be greater than dimension of neighbourhood complex*.

*Proof*. Let assume the highest degree vertex in *G* is *v* with degree *k*. These *k*-vertices will be (*k* − 1)-simplex in corresponding neighbourhood complex *N*(*G*) and *dim*(*N*(*G*)) = *k* − 1.

Let, *dim*(*C*(*G*)) > *dim*(*N*(*G*)) and *dim*(*C*(*G*)) = *k* ⇒ ∃ a clique *c* of size *k* + 1 in *G* and every vertex of this clique have degree exactly *k*. Here two conditions arise:

- Either clique *c* is isolated from remaining graph.
- Or the given graph *G* is a complete graph on *k* + 1 vertices.

In both the conditions contradiction will arise. Hence, dimension of clique complex cannot be greater than dimension of neighbourhood complex.

Facets of a simplicial complex are the maximal simplices, and the study of facets can give us deeper insights into the simplicial complex. In the *q*-analysis of clique and neighbourhood complex, the study of their facets can give us groups of social-ecological units that have special kinds of interactions with in the system. Moreover, the *q*-connectivity of these simplices is also different from the other simplices. In the next theorem, we can see how facets of a clique and neighbourhood complex participate in the *q*-connectivity of the complex.

**Theorem 2.3** *In a simplicial complex* Δ *of dimension K, every facet of* Δ *contributed as a individual q*-*connected component at q* = *dim*(*facet*)-*level*.

*Proof*. Let *σ* be a facet of dimension *k*, where 0 ≤ *k* ≤ *K*. Let assume *σ* does not contribute as a individual *q*-connected component at the *q*^*th*^-level, where *q* = *dim*(*σ*) = *k*. At *q* = *k* level the dimension of all the simplices are greater than or equal to *k*. Which implies that ∃ at least a simplex *τ* other than *σ* which is *k*-near with *σ*. This further implies that *σ* ⊂ *τ*. Which is a contradiction.

### 3.2 Simmelian brokerage and topological dimension

In social ecological networks interactions of social and ecological units can be used to interpretate the meaning of social-ecological capital. In order to estimate the social-ecological capital of vertices (ecological and social units) in the network, Simmelian brokerage *B*_*i*_ for each 0-simplex *i* = {1, 2, …, 22} has been calculated. According to [31], for a given 0-simplex *i*, simmelian brokerage “captures opportunities of brokerage amongst otherwise disconnected cohesive groups of contacts”. Quantitatively, *B*_*i*_ is determined by the vertex’s efficiency *E*_*i*_, For a given vertex *i* which has the neighbours count *n*_*i*_ in the considered group of neighbours *N*_*i*_ is given by:

$$\begin{array}{c} B_{i}=n_{i}-\left(n_{i}-1\right)E_{i},\text{where}\;E_{i}\;\text{is}\;\text{given}\;\text{by} \end{array}$$

$$\begin{array}{c} E_{i}=\frac{1}{n_{i}\left(n_{i}-1\right)}\sum_{m\in N_{i}}\sum_{n\in N_{i}}\frac{1}{d_{mn}} \end{array}$$

Here, *d*_*mn*_ is the shortest path distance between vertex *m* and *n* by omitting the vertex *i*.

Topological dimension of every 0-simplex in a simplicial complex *K* is given by the total number of simplices in which 0-simplex participates. For expressing this, a vector of dimension equal to the dim(K)+1 denoted by $Q_{q}^{i}$ has been introduced for every 0-simplex *i* of the given simplicial complex *K* [39]. Every component of the node’s vector $Q_{q}^{i}$ gives the number of simplices of dimension *q*, in which 0-simplex *i* participates. The summation of every component of vector $Q_{q}^{i}$ gives us the topological dimension of 0-simplex *i*. For example, node’s vector corresponding to the 0-simplex *E*1 is given by $Q_{q}^{E1}=[0\;3\;4\;1]$. Hence, the topological dimension of *E*1 is 0 + 3 + 4 + 1 = 8.

**Fig 3:**
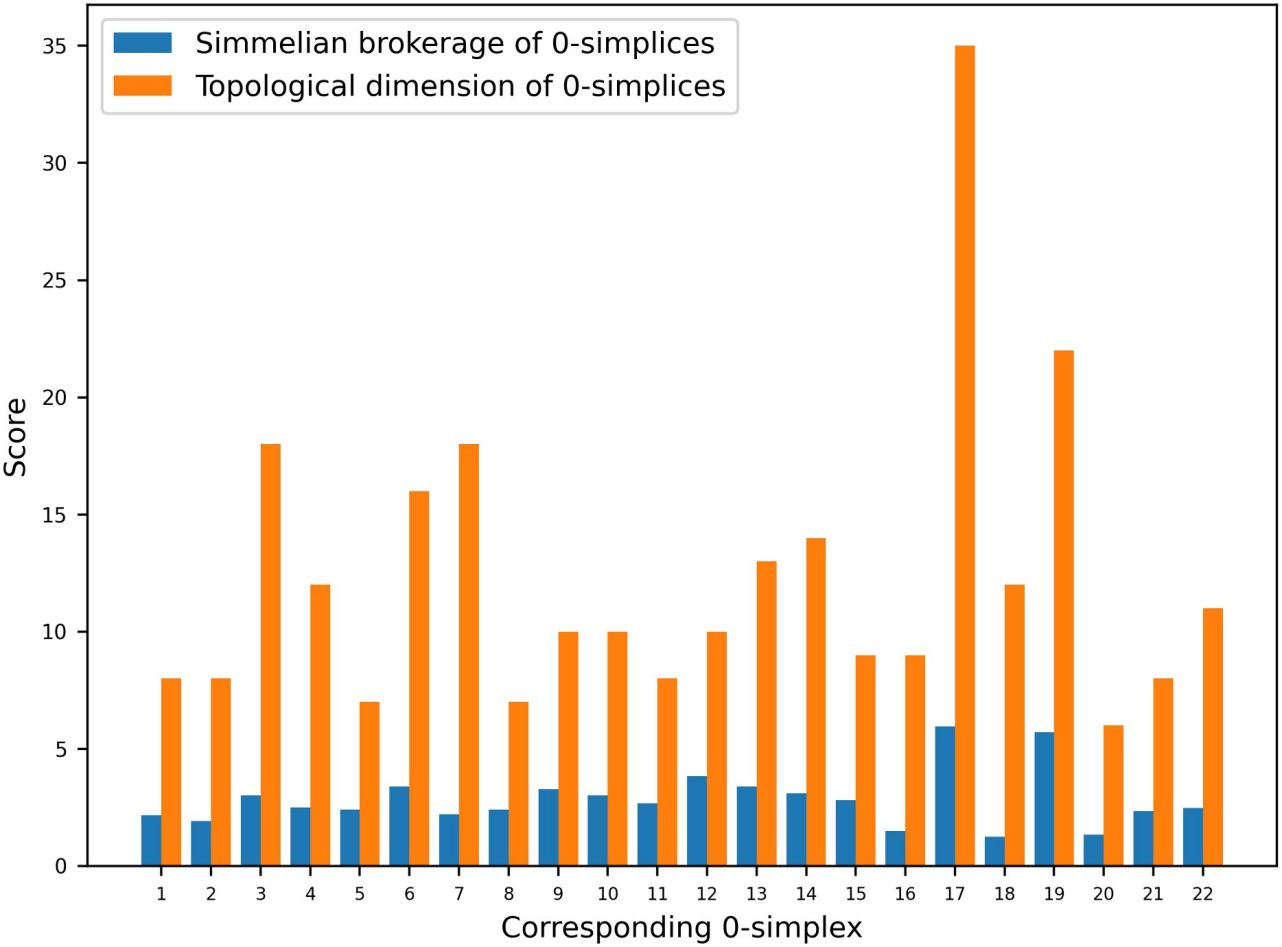
Plot of simmelian brokerage and topological dimension of clique complex *C*(*N*_*M*_).

In the Fig 3. it can be observed that for the 0-simplex *S*3 the value of simmelian brokerage is highest which is 5.95. The corresponding topological dimension of vertex *S*3 can be calculated by its node’s vector which is given as $Q_{q}^{S3}=[5\;17\;12\;1]$, 5+17+12+1 = 35. It signifies that the vertex *S*3 has the highest social-ecological capital, which further suggest that vertex *S*3 can be act as broker in communities. Among the ecological units *E*3 has the highest value of topological dimension, and it can be concluded that *E*3 participated in the highest number of simplices formed in the clique complex of *C*(*N*_*M*_).

### 3.3 Analysis of q-nearness in clique complex *C*(*N*_*M*_)

In the Q-analysis, a simplicial complex *K* is studied at different connectivity levels, designated by *q* and it varies from 0 ≤ *q* ≤ *dim*(*K*). At these different connectivity levels, we can understand the formation of higher-order interactions in any given system represented by a simplicial complex. The dimension of the constructed clique complex *C*(*N*_*M*_) of the Madagascar system is 3, hence one can study the structural details of the Madagascar system for different values of *q*, where 0 ≤ *q* ≤ 3.

**0-nearness**: Two given simplices are said to be 0-near if they share a common 0-dimensional face. Hence, one of the simplex must have dimension greater than 0. If the dimension of both the simplices are one, then it is the same as the graph-theoretic adjacency (two edges are adjacent if they share a common vertex). By the first and second structural vectors given in Table 2, the components corresponding to the *q* = 0 are 1 and 121 respectively. Which implies that all the simplices of dimension 0 or more (which are 121 in counting) are in single 0-connected component. Which further suggests that at *q* = 0 level structure is the same as a graph-theoretic network, as the connectivity is only depending upon the sharing of 0-dimensional simplex which is a vertex.

**Table 2:** Table showing the q-analysis of the simplicial complex (neighbourhood complex). The first column is the different connectivity levels of the complex. The second column is the number of *q*-connected components at each level, while the third column reports the names of the simplices making up each q-connected component.

*q*	*Q*_*q*_	Components
11	1	{*S*_3_}
10	1	{*S*_3_}
9	1	{*S*_3_}
8	2	{*S*_3_}, {*S*_5_}
7	2	{*S*_3_}, {*S*_5_}
6	2	{*S*_3_}, {*S*_5_}
5	2	{*S*_3_}, {*S*_5_}
4	4	{*S*_3_}, {*S*_5_}, {*S*_1_}, {*S*_8_}
3	6	{*S*_3_}, {*S*_1_}, {*S*_8_}, {*S*_2_}, {*S*_4_}, {*S*_5_}
2	4	{*S*_1_, *S*_3_, *S*_4_, *S*_5_, *S*_6_}, {*S*_2_}, {*S*_7_}, {*S*_8_}
1	2	{*S*_1_, *S*_2_, *S*_3_, *S*_4_, *S*_5_, *S*_6_}, {*S*_7_, *S*_8_}
0	1	{*S*_1_, *S*_2_, *S*_3_, …, *S*_8_}

**1-nearness**: If the dimensions of the two simplices are at least 1, then we can discuss their 1-nearness. By the first and second structural vectors given in Table 2, the components corresponding to the *q* = 1 are *Q*_1_ = 5 and *n*_1_ = 99 respectively, *Q*_1_ denotes the number of 1-connected components at this level. Out of these five 1-connected components, 3 are facets of dimension 1, which contribute as individual components at the *q* = 1 level, according to Theorem 3.5. Remaining non-facets simplices of dimension 1 can be 1-near with simplices of dimension 2 and 3, and always form an interaction type of triangle or tetrahedral as for 1-nearness, it should be contained in the simplices of a higher dimension. These interactions can be studied at the *q* = 2 or 3 level (as these 1-simplices must be the face of any 2-simplex or 3-simplex).

Simplices of dimension 2 can be 1-near with simplices of dimension 2 and 3. First, we will see the 1-nearness between simplices of dimension 2. After observing most of the patterns, they can be classified into two types:

(A) If two social units are utilising more than one same ecological patch, they are considered to be 1-near and can be seen as a part of “1-connected component.”
(B) Higher-order interactions of order 2 between ecological units are also part of the 1-connected component.

**Fig 4:**
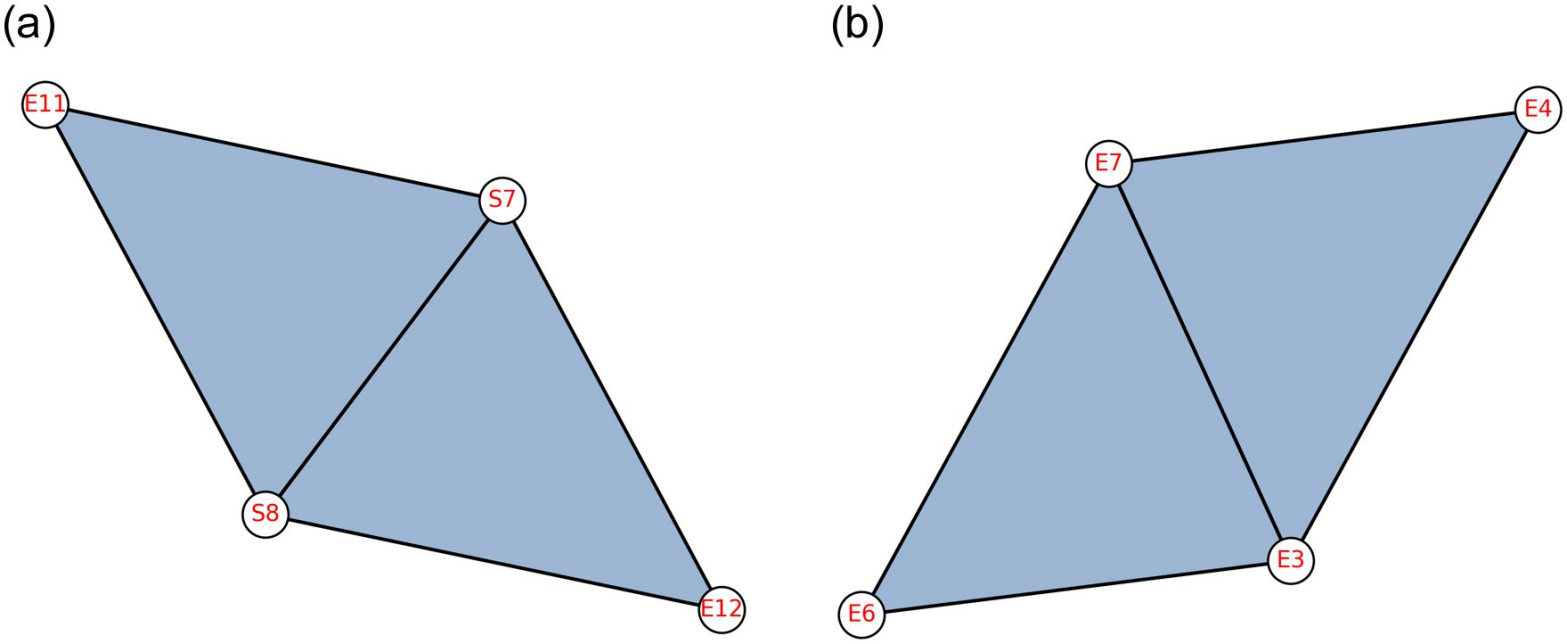
(a) 1-nearness in the simplices of dimension 2 formed by the combination of social and ecological units (b) 1-nearness in the simplices of dimension 2 formed by the ecological units alone.

In Fig 4a, it can be seen that social units *S*7 and *S*8 are utilising ecological patches *E*12 and *E*11, due to which *S*7 and *S*8 are socially connected; hence, these two higher-order interactions {S7, S8, E12} and {S7, S8, E11} are 1-near to each other. On the other hand, higher-order interactions between ecological units represented by simplices {*E*6, *E*7, *E*3} and {*E*4, *E*7, *E*3} are also 1-near to each other by sharing two common ecological units.

**Fig 5:**
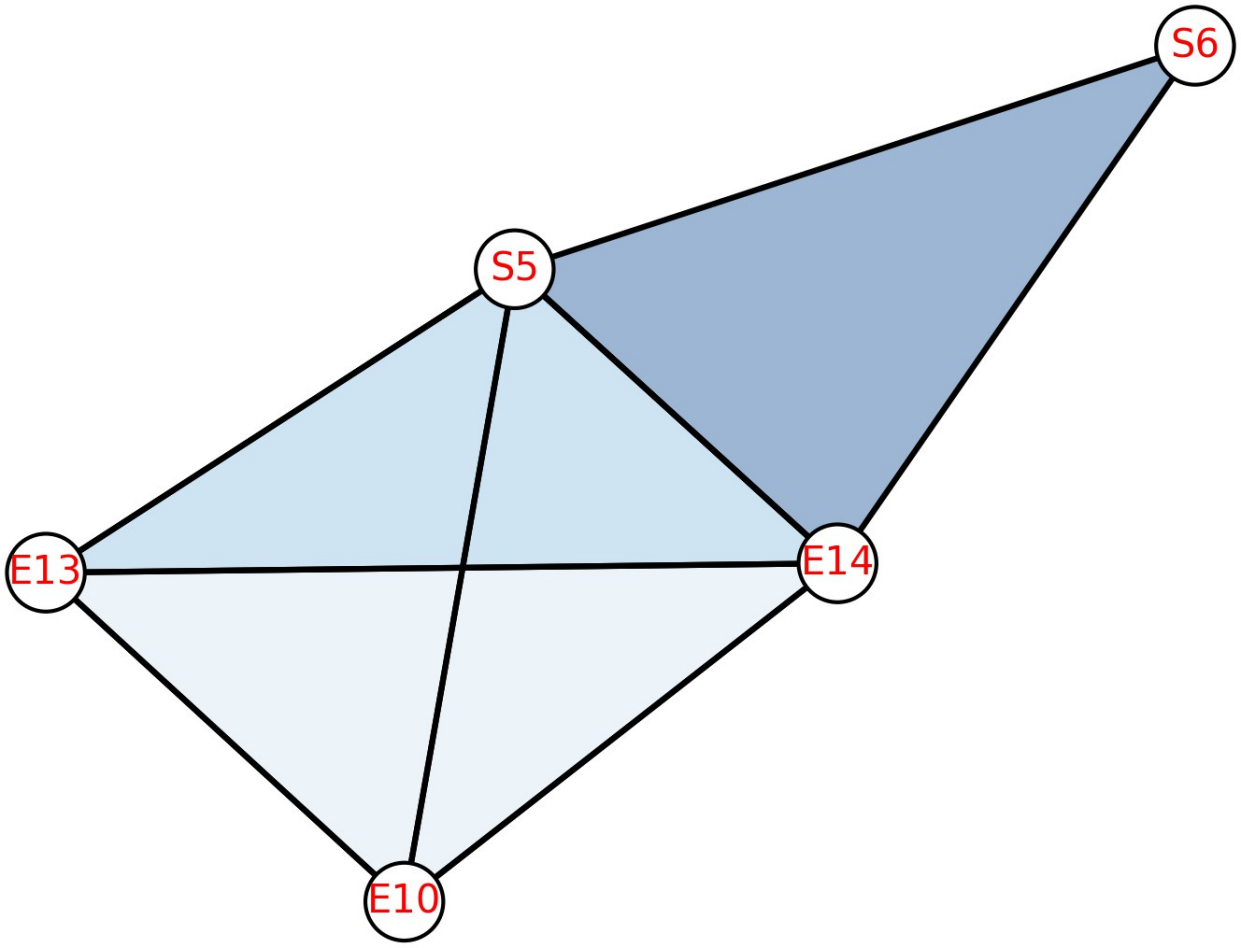
1-nearness between facet of dimension 2 and a simplex of dimension 3. Both the simplices shared a 1-dimensional face {S5, E14} with each other.

Simplices of dimension 2 can also be 1-near with simplices of dimension 3 by sharing a face of dimension 1. Here, also two different patterns are observed, either the simplex of dimension 2 is contained in the simplex of dimension 3, or it will share only one dimensional face with the 3-simplex. In the second case, the 2-simplex will be the facet of the simplicial complex (as it will not be contained in any other simplex). *C*(*N*_*M*_) contains 10 facets of dimension 2, hence 1-nearness can be observed between facets of dimension 2 and 3-simplices of *C*(*N*_*M*_). An example of such interaction is given Fig 5. in which a facet {*S*6, *S*5, *E*14} is 1-near with a 3-simplex {*S*5, *E*14, *E*10, *E*13}.

**2-nearness**: Two simplices can be 2-near to each other, if at least one of them have the dimension greater than 2 (as simplices of dimension 2 can be 2-near if they are same simplices). From the first and second structural vector, number of 2-connected components in *C*(*N*_*M*_) are 17 and simplices of dimension greater than and equal to two are 44. Hence, two pattern of 2-nearness is observed in *C*(*N*_*M*_).

(A) Between simplices of dimension 2 and 3.

(B) Between the simplices of dimension 3.

**Fig 6:**
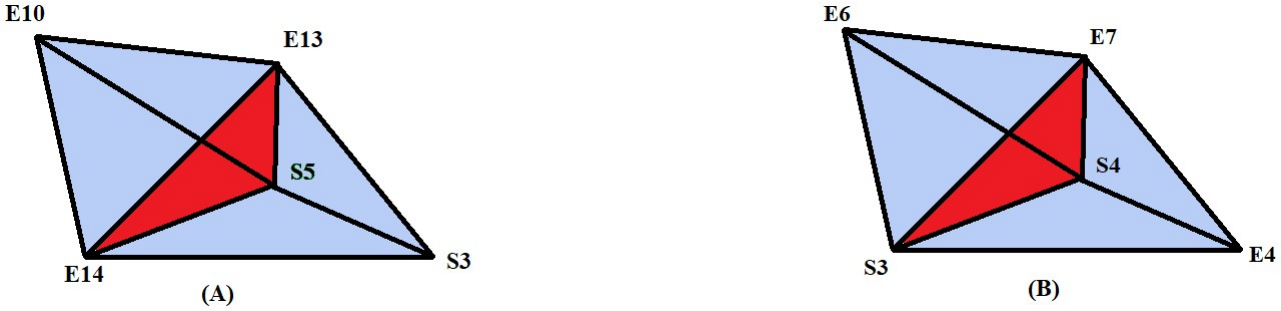
2-nearness between two simplices of dimension 3, red shaded simplex is the common to both the simplices. In (A) 3-simplices {E10, E13, E14, S5} and {E13,E14,S5,S3} shared a common 2 dimensional simplex {E13, E14, S5}. In (B) 2-simplex {S3, E7, S4} is shared by two 3-simplices.

A simplex of dimension 2 can be 2-near with a simplex of dimension 3, if it is a face of the dimension 3 simplex. By which we can directly conclude that facets of dimension 2 cannot be 2-near in the *C*(*N*_*M*_) as they cannot be a face of any other bigger simplex. Hence, these interactions of order 2 represented by facets are individual 2-connected components at *q* = 2 level. 2-nearness can also be defined between simplices of dimension 3. It will be formed by simplices of dimension 3, which share only a 2-dimensional face. An example of this type of interaction is given in Fig 6.

## 4 Q-analysis of neighbourhood complex of Madagascar network

In this section, we have constructed two neighbourhood complexes from the pairwise network of Madagascar *N*_*M*_. One corresponds to the social units of *N*_*M*_, which is denoted by *N*_*S*_(*N*_*M*_), and the other one corresponds to the ecological units of *N*_*M*_, which is denoted by *N*_*E*_(*N*_*M*_).

### 4.1 Analysis of *q*-nearness in neighbourhood complex of social units *N*_*S*_(*N*_*M*_)

**Fig 7:**
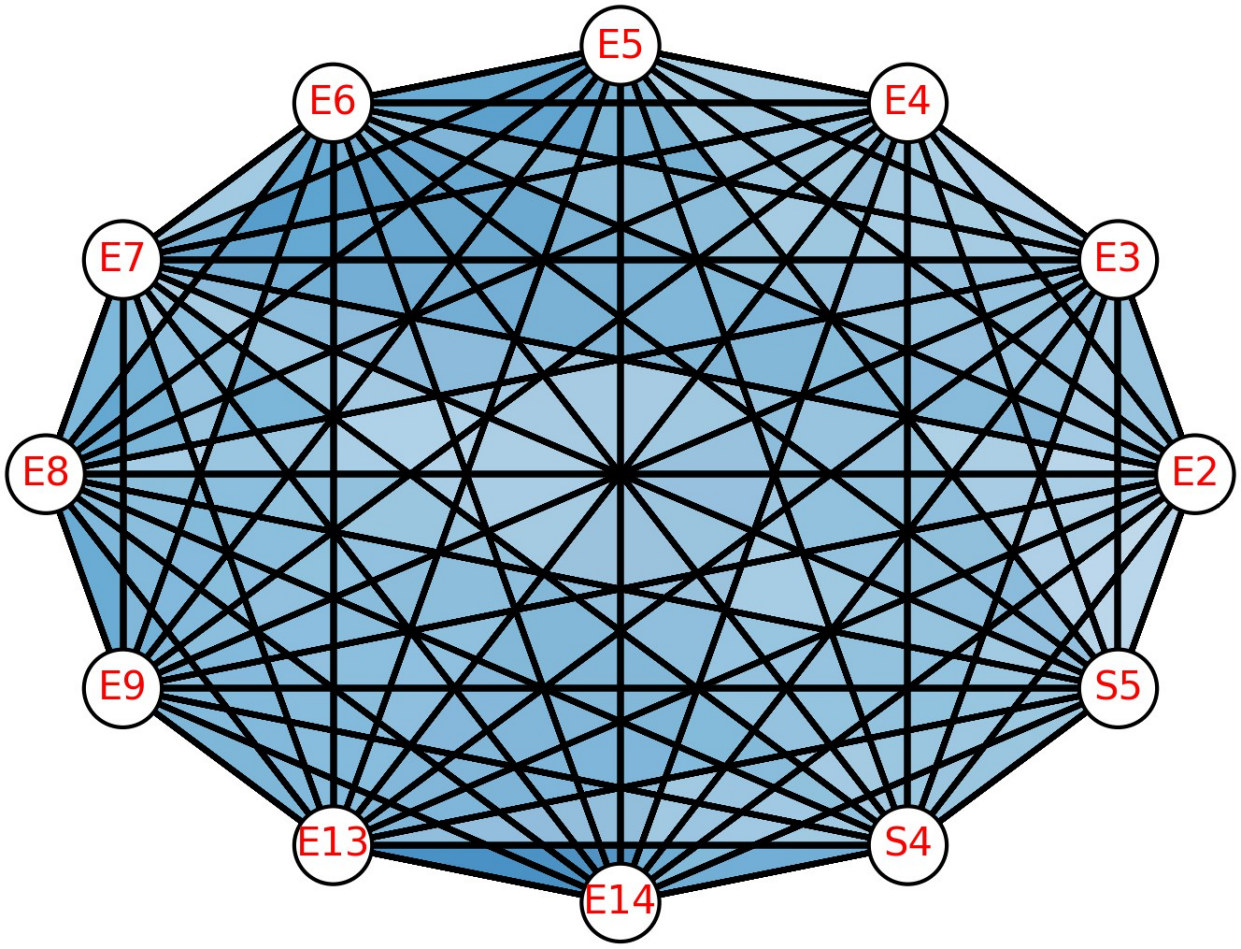
Simplex *S*_3_ of dimension 11 corresponding to social unit *S*3, whose structure is given as: *S*_3_ = {E2, E3, E4, E5, E6, E7, E8, E9, E13, E14, S4, S5}, it consist 12 vertices.

**Fig 8:**
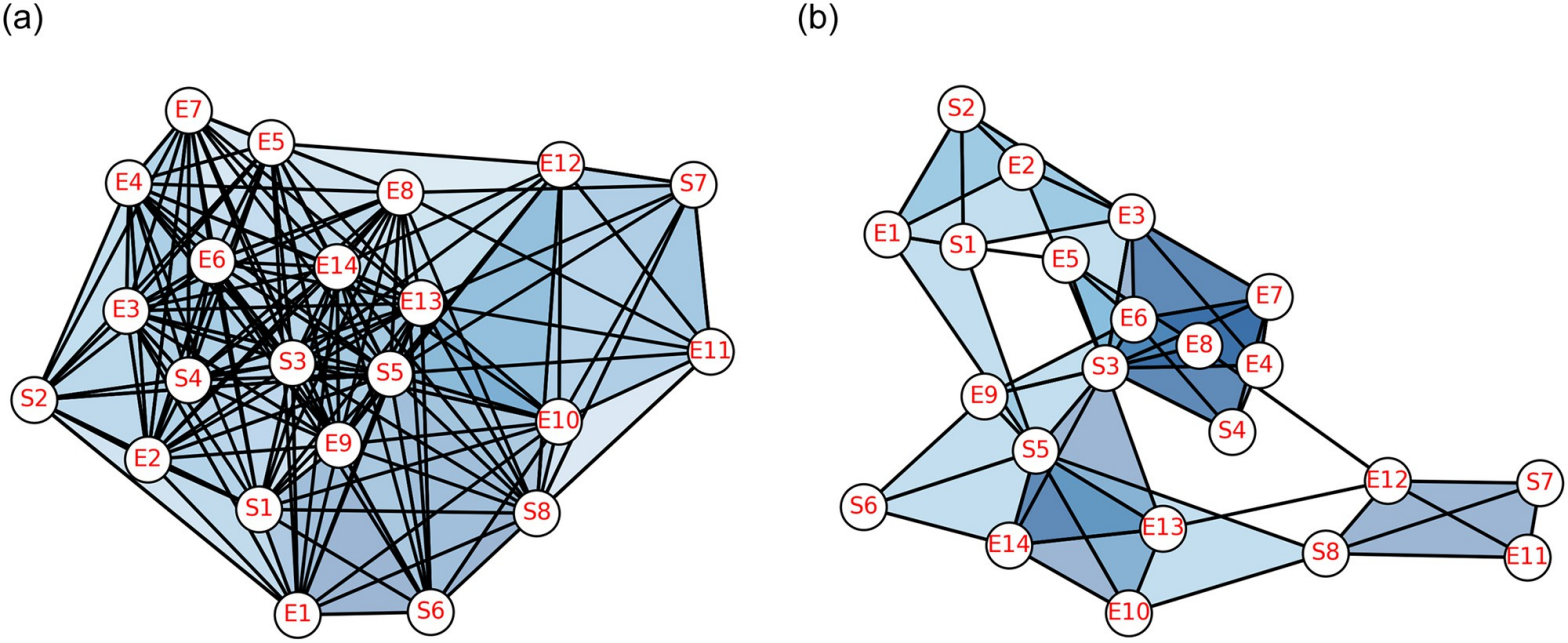
(a) Neighbourhood complex of pairwise network *N*_*M*_ of dimension 11 (b) Clique complex of pairwise network *N*_*M*_ of dimension 3.

The neighbourhood complex of the given Madagascar network *N*_*M*_ has been constructed for social units and it contains the simplices corresponding to social units only. Each social unit, *Si*, has been represented as a simplex of the social and ecological units for which *Si* has been a common neighbour. Corresponding to 8 social units, 8 simplices of different dimensions have been formed and represented by *S*_*i*_. The highest simplex of dimension 11 has been formed corresponding to the social unit *S*3, which implies that *S*3 has the highest number of neighbours in the pairwise network of Madagascar and is given in Fig 7. Every social unit has at least 3 neighbours in the *N*_*M*_, which implies that the minimum dimension of any simplex in a neighbourhood complex is 2. By doing this, simplices that formed corresponding to different social units will reveal the formation of different social ties depending upon the social and ecological units. The neighbourhood complex of of social units *N*_*S*_(*N*_*M*_), contains simplices corresponding to social units only *Si*, hence it is a sub complex of the neighbourhood complex *N*(*N*_*M*_) given in Fig 8a.

As the structure suggests, *S*_3_ contains 10 different ecological units as their vertices and 2 social units. It implies that *S*3 is adjacent to 10 ecological and 2 social units. From the ecological point of view, *S*3 utilises most of the ecological units or patches, and due to this, {*S*3, *S*4} and {*S*3, *S*5} are socially connected in the original pairwise network *N*_*M*_, as they both also uses the same ecological patches.

The structure of neighbourhood complex can be seen as *N*_*S*_(*N*_*M*_) = {*S*_1_, *S*_2_, …, *S*_8_} along with all its faces and the Q-analysis of *N*_*S*_(*N*_*M*_) has been given in Table 2. At the *q* = 0 level, all the simplices lie in the single component, which implies that each simplex shares at least one ecological or social unit with any of the simplex. Hence, they are all in the same component and further 0-connected to each other. Simplices *S*_3_ and *S*_1_ share the highest number of social-ecological units, which are 3, hence they are 2-connected to each other. No connection of any type has been observed between simplices of dimension greater than 2, which further suggests that connectivity levels greater than 2 contain only singleton components.

### 4.2 Analysis of *q*-nearness in neighbourhood complex of ecological units *N*_*E*_(*N*_*M*_)

Corresponding to each ecological unit *Ei* a simplex *E*_*i*_ has been constructed by the vertices of *N*_*M*_ which have the common neighbour *Ei*. The largest simplex *E*_3_ is of dimension 6 and its structure is given as *E*_3_ = {*E*2, *E*4, *E*6, *E*7, *S*1, *S*2, *S*3}, it consist of 4 ecological and 3 social units from pairwise network *N*_*M*_. Simplex *E*_3_ which is a simplex corresponding to the ecological unit *E*3 also has largest area among all the ecological patches of *N*_*M*_.

The structure of neighbourhood complex of ecological units is given as *N*_*S*_(*N*_*M*_) = {*E*_1_, …, *E*_14_} along with all its faces and its q-analysis is given in Table 3. At *q* = 0 and *q* = 1 level, all the simplices lie in the single component, which implies that all *E*_*i*_s are 1-connected to each other. As value of *q* are increasing the number of *q*-connected components are also increasing. At *q* = 3 level, one can observe the sudden increase in the number of *q*-connected components, it implies that very few simplices in *N*_*E*_(*N*_*M*_) sharing more than 3 ecological/social units with each other and participating as individual 3-connected component. Only simplices which form 3-connected component are *E*_6_ and *E*_4_. On *q* = 4 onward, no simplex is sharing a face of dimension 4 or more with any other. Highest *q*-near value in this complex is 3, as *E*_6_ and *E*_4_ are 3-near to each other.

**Table 3:** Table showing the *q*-analysis of the simplicial complex (neighbourhood complex). The first column is the different connectivity levels of the complex. The second column is the number of q-connected components at each level, while the third column reports the names of the simplices making up each q-connected component.

*q*	*Q*_*q*_	Components
6	1	{*E*_3_}
5	2	{*E*_3_}, {*E*_6_}
4	7	{*E*_7_}, {*E*_9_}, {*E*_12_}, {*E*_13_}, {*E*_14_}, {*E*_3_}, {*E*_6_}
3	12	{*E*_6_, *E*_4_}, {*E*_1_}, {*E*_2_}, {*E*_3_}, {*E*_5_}, {*E*_7_}, {*E*_8_}, {*E*_9_}, {*E*_10_}, {*E*_12_}, {*E*_13_}, {*E*_14_}
2	5	{*E*_1_, *E*_3_, …, *E*_14_}, {*E*_2_}, {*E*_10_}, {*E*_11_}, {*E*_12_}
1	1	{*E*_1_, *E*_2_, *E*_3_, …, *E*_14_}
0	1	{*E*_1_, *E*_2_, *E*_3_, …, *E*_14_}

With the help of the *q*-analysis of the neighbourhood complex of social units and ecological units, one can identify the largest dimensional simplices and their corresponding social and ecological units, which are *S*3 and *E*3 respectively. Corresponding to *S*3 and *E*3, there exist simplices *S*_3_ and *E*_3_ respectively, which contain their neighbours. Moreover, we can understand the participation of their neighbours in the formation of other simplices at different *q*-levels. This can be helpful in understanding the interactions of social-ecological units in Madagascar, as the depth of material transfer, intricately linked to the size and proximity of patches to social units, governs the extraction and utilisation of resources, ushering in a nuanced perspective on resource dynamics. The gamut of resource utilisation, spanning cattle herding, timber harvesting for charcoal production and fuel, and agriculture, are the recipient-oriented interactions that maintain the complex yet delicate balance shaping Madagascar’s social ecological system as investigated in the present study [40]. The ecological unit *E*3, which has the highest number of connections among the ecological units, can be treated as a source of resources.

## 5 Results and calculations

In this section, all the results obtained from the *Q*-analysis for both simplicial complexes have been discussed. An analysis of all three structural vectors for both the constructed simplicial complexes has also been done.

- The pairwise social-ecological network of southern-Madagascar consist 22 vertices (14-ecological and 8 social vertices).
- The relation between the vertices is: generating and managing ecological resources in the same forest patch. By this relation, two ecological units can be related to each other, whereas two social units can also be related to each other depending on their utilization of the same forest patch.
- The overarching nature of this relationship also involves the social-ecological interactions of the given network.
- Clique complex of the given pairwise network has been constructed by taking cliques of *N*_*M*_ as simplices of appropriate order.
- Neighbourhood complex has been constructed by making simplices corresponding to each vertex by its neighbours.
- The constructed clique complex is of dimension 3, whereas the dimension of neighbourhood complex is 11. Which further implies that clique complex have 4 levels of connectivity whereas neighbourhood complex have 12 levels of connectivity.
- Q-analysis of the clique complex and neighbourhood complex of *N*_*M*_ has been done and all the structural vectors have been calculated and given in Table 4.

**Table 4:** Different structural vectors of clique complex and neighbourhood complex of *N*_*M*_. The dimension of all the structural vectors of clique complex is 4, whereas dimension of all the structural vectors of neighbourhood complex is 12.

	Clique complex *C*(*N*_*M*_)	Neighbourhood complex *N*(*N*_*M*_)
**Dimension of simplicial complex**	3	11
**First structural vector *Q*_*q*_**	[7 17 5 1]	[1 1 1 2 2 3 4 10 13 5 1 1]
**Second structural vector *n*_*q*_**	[7 44 99 121]	[1 1 1 2 2 3 4 11 19 22 22 22]
**Third structural vector** ${\bar{Q}}_{q}$	[0 0.61 0.95 0.99]	[0 0 0 0 0 0 0 0.09 0.31 0.77 0.95 0.95]

**Fig 9:**
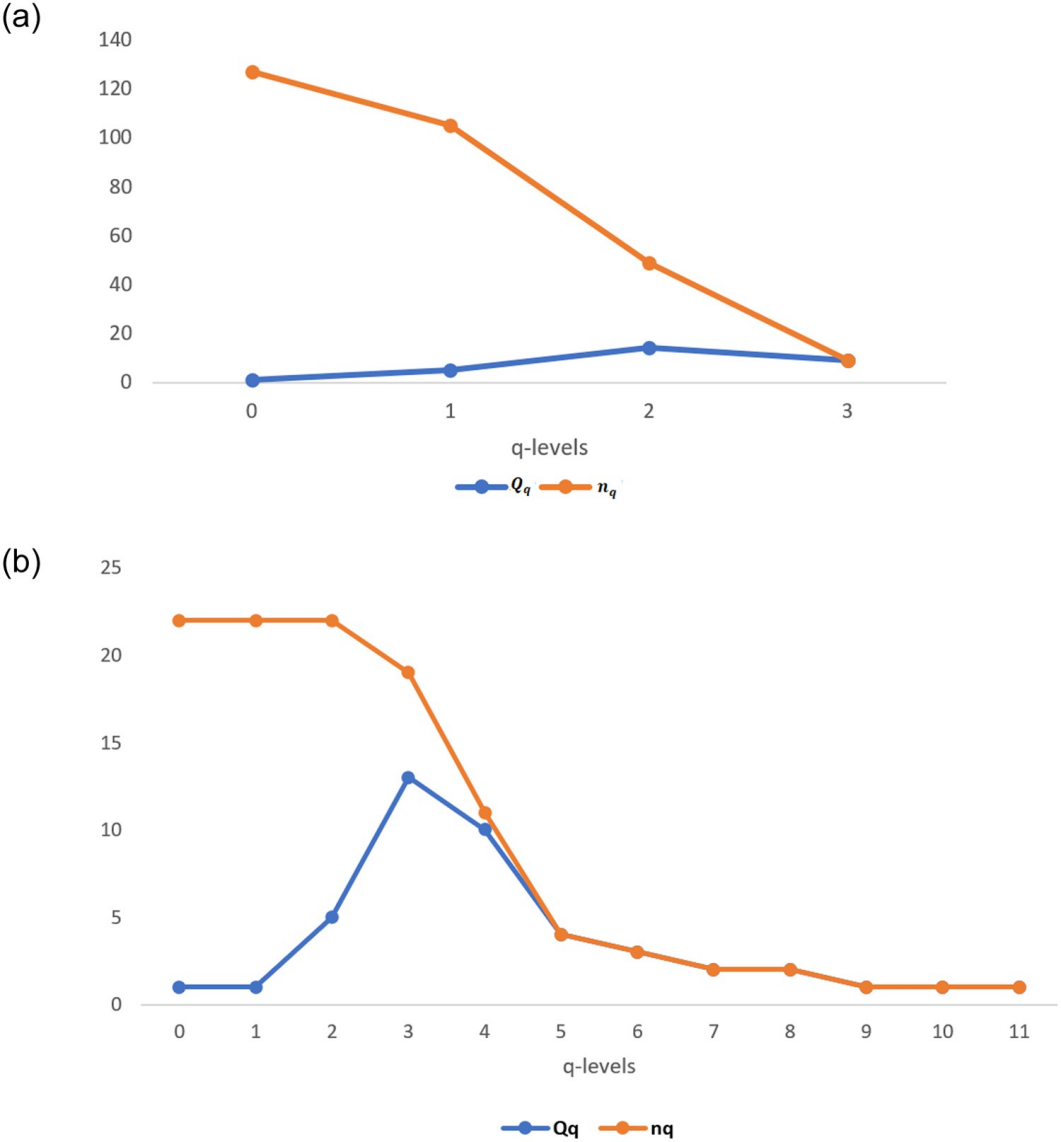
Plot of first and second structural vectors of (a) Clique complex and (b) Neighbourhood complex.

The first structural vector of clique complex *C*(*N*_*M*_) is *Q* = [7 17 5 1], every entry of this vector can be seen as a sum of two classes one contains the number of connected components without facets and other one which contains the facets of dimension q. For example, *Q* can be written as *Q* = [0 + 7 3 + 14 2 + 3 1 + 0], here 7, 14, 3, and 0 are the facets of dimension 3, 2, 1 and 0 respectively of *C*(*N*_*M*_). Total facets of *N*(*N*_*M*_) are 19 which contribute as individual *q*-connected component at appropriate *q*-level. Moreover, every facet of *dim* ≥ 2 contain one ecological unit and one social unit at least. But there are only three higher-order interactions which contains only ecological units are; {*E*3, *E*4, *E*7}, {*E*3, *E*6, *E*7}, {*E*10, *E*13, *E*14} and all the other higher-order interactions contain one ecological unit and one social unit atleast. Comparison plot of first and second structural vectors are given in Fig 9. for both the complexes.

The overlap of values in Fig 9. for some *q*-levels like 7, 8, 9, 10, and 11 in the neighbourhood complex and for level 3 in the clique complex indicate that at these *q*-levels, structure is disconnected.

The third structural vector ${\bar{Q}}_{q}$ of *C*(*N*_*M*_) increases as *q*-connected components in *Q*_*q*_ decreases, and one can observed that ${\bar{Q}}_{q}\to 1$ as $\frac{Q_{q}}{n_{q}}\to 0$. The ratio of $\frac{Q_{q}}{n_{q}}$ is signifies the *q*-connected components per simplex of any simplicial complex, as going on left to right in the calculated structural vectors the ratio tends to zero which signifies the increasing value of ${\bar{Q}}_{q}$. Moreover, each component of ${\bar{Q}}_{q}$ tells us the percentage of simplices in the simplicial complex which are *q*-connected.

- Highest eccentricity simplex in the neighbourhood complex is $\sigma_{v_{17}}=S_{3}=\{E2,E3,E4,E5,E6,E7,E8,E9,E13,E14,S4,S5\}$ with value 0.461. Many simplices have an eccentricity value of 0 in both simplicial complexes, which implies that these simplices are totally integrated into the structure and have zero individuality.
- In Table 5, simplices with the highest eccentricity values are given for both the constructed simplicial complexes. Simplex *S*_3_ from the neighbourhood complex has the highest value of eccentricity, as it is highest dimensional simplex and participated in the community structures with most of the simplices.

**Table 5:** List of top simplices with eccentricity values in both simplicial complexes.

	Clique complex *C*(*N*_*M*_)	Neighbourhood complex *N*(*N*_*M*_)
**Top eccentricity simplex**	{E5,S1}; {E12,E13}; {E10,S5,S8}	{E2, E3, E4, E5, E6, E7, E8, E9, E13, E14, S4, S5}; {E9, E14, S5};
**Corresponding values**	0.50, 0.50, 0.33	0.46, 0.40

## 6 Conclusions

In this work, a structural study of the rural agricultural system of southern Madagascar has been done by constructing clique and neighbourhood complexes from the available pairwise social-ecological network. Using simplicial complex representation of this network, we investigated the in-depth topology of the given Madagascar network *N*_*M*_. With the help of Q-analysis, which gives us an understanding of structure at different topological levels, we gain deeper insights into the structure studied. All three structural vectors, along with the node’s vector, which leads us to the topological dimension, have been calculated and compared for both simplicial complexes. Interesting results about the participation of facets of the clique complex at different *q*-levels have been studied and given by the theorem. Moreover, some interesting results on the dimension of neighbourhood complex and clique complex have been compared on the basis of the graph type from which they originated.

An in-depth analysis of *q*-nearness in clique complexes gave us an idea about how higher-order interactions are sharing social and ecological units in the form of *q*-faces with each other. This research delves into the intricate fabric of Madagascar’s social ecological system, revealing the multifaceted interactions between diverse units that extend beyond mere pairwise associations. Central to this investigation is the concept of information, referring to the resources harnessed by various anthropogenic units from forests and agricultural lands. Employing input-output analysis and ecological network analysis, the flow of materials can be quantified, thus portraying the intricate roles played by different nodes within the social and ecological units. Remarkably, social units emerge as the receivers of resources (sinks), while ecological units act as the sources, manifesting a bidirectional exchange that defies traditional unidirectional energy-flow models.

The concept of ‘*q*-nearness’ has illuminated higher-order interactions, showcasing how diverse units—both social and ecological—collaborate and share resources in ways that challenge conventional models of energy flow. Our work also sheds light on the perpetual symbiotic relationship existing between human communities and their natural environment, highlighting the vital bidirectional exchange of resources that defines this dynamic social-ecological landscape. This study contributes to a more holistic comprehension of the intricate web of interactions from a social-ecological perspective, thus providing valuable insights for sustainable practices and future research in both social and ecological domains.

In order to further strengthen our methodology, future works require a more detailed study of the higher-order interactions present in social-ecological networks, corresponding to every possible relationship exists in the social-ecological system. To properly define the simplices of a simplicial complex, a well-defined overarching relation is required, by which the interactions of lower-order as well as higher-order of units can be defined. Analysis of each and every relation in a large clique and neighbourhood complex is not easy as the *q*-levels will increase, which makes it difficult to understand how social and ecological units are shared among the systems and can be seen as a limitation of this framework. Another opportunity that can strengthen this work is the availability of higher-order data, which will definitely help in the detailed study of the structure of the system, as constructing a simplicial complex from a pairwise network always required an overarching relation to the defined simplices of the system, which is not possible every time.

## Supporting information

S1 File Data for Madagascar network. (CSV)
